# Illicit Trade of Prescription Medications Through X (Formerly Twitter) in Japan: Cross-Sectional Study

**DOI:** 10.2196/54023

**Published:** 2024-05-28

**Authors:** Hayase Hakariya, Natsuki Yokoyama, Jeonse Lee, Arisa Hakariya, Tatsuki Ikejiri

**Affiliations:** 1 Interfaculty Institute of Biochemistry University of Tuebingen Tuebingen Germany; 2 Laboratory for Human Nature, Cultures and Medicine Shiga Japan; 3 Department of Pharmacy Chubu Tokushukai Hospital Okinawa Japan; 4 School of Medicine Kyoto Prefectural University of Medicine Kyoto Japan; 5 General Hospital Minami Seikyo Hospital Aichi Japan

**Keywords:** illegal trading, pharmacovigilance, social networking service, SNS, overdose, social support, antipsychotics, Japan, prescription medication, cross-sectional study, prescription drug, social networking, medication, pharmaceutical, pharmaceutical drugs, Japanese, psychiatric, support

## Abstract

**Background:**

Nonmedical use of prescription drugs can cause overdose; this represents a serious public health crisis globally. In this digital era, social networking services serve as viable platforms for illegal acquisition of excessive amounts of medications, including prescription medications. In Japan, such illegal drug transactions have been conducted through popular flea market applications, social media, and auction websites, with most of the trades being over-the-counter (OTC) medications. Recently, an emerging unique black market, where individuals trade prescription medications—predominantly nervous system drugs—using a specific keyword (“Okusuri Mogu Mogu”), has emerged on X (formerly Twitter). Hence, these dynamic methods of illicit trading should routinely be monitored to encourage the appropriate use of medications.

**Objective:**

This study aimed to specify the characteristics of medications traded on X using the search term “Okusuri Mogu Mogu” and analyze individual behaviors associated with X posts, including the types of medications traded and hashtag usage.

**Methods:**

We conducted a cross-sectional study with publicly available posts on X between September 18 and October 1, 2022. Posts that included the term “Okusuri Mogu Mogu” during this period were scrutinized. Posts were categorized on the basis of their contents: buying, selling, self-administration, heads-up, and others. Among posts categorized as buying, selling, and self-administration, medication names were systematically enumerated and categorized using the Anatomical Therapeutic Chemical (ATC) classification. Additionally, hashtags in all the analyzed posts were counted and classified into 6 categories: medication name, mental disorder, self-harm, buying and selling, community formation, and others.

**Results:**

Out of 961 identified posts, 549 were included for analysis. Of these posts, 119 (21.7%) referenced self-administration, and 237 (43.2%; buying: n=67, 12.2%; selling: n=170, 31.0%) referenced transactions. Among these 237 posts, 1041 medication names were mentioned, exhibiting a >5-fold increase from the study in March 2021. Categorization based on the ATC classification predominantly revealed nervous system drugs, representing 82.1% (n=855) of the mentioned medications, consistent with the previous survey. Of note, the diversity of medications has expanded to include medications that have not been approved by the Japanese government. Interestingly, OTC medications were frequently mentioned in self-administration posts (odds ratio 23.6, 95% CI 6.93-80.15). Analysis of hashtags (n=866) revealed efforts to foster community connections among users.

**Conclusions:**

This study highlighted the escalating complexity of trading of illegal prescription medication facilitated by X posts. Regulatory measures to enhance public awareness should be considered to prevent illegal transactions, which may ultimately lead to misuse or abuse such as overdose. Along with such pharmacovigilance measures, social approaches that could direct individuals to appropriate medical or psychiatric resources would also be beneficial as our hashtag analysis shed light on the formation of a cohesive or closed community among users.

## Introduction

Nonmedical use of prescription drugs, which can lead to overdose (OD), is a serious public health crisis in worldwide [1-4]. The US Centers for Disease Control and Prevention reported that 70,630 people died from OD in 2019; of them, 49,860 and 9711 deaths were caused by opioids and benzodiazepines, respectively [5]. In Japan, benzodiazepines such as flunitrazepam, and over-the-counter (OTC) antipyretic analgesics, antitussives, and cold medicines are reported as major causes of OD [6-9].

In this contemporary digital era, social networking services (SNSs) are emerging as viable platforms to share health-related information among individuals [10-12]. Despite its illicitness, individuals have been acquiring excessive amounts of medications [13-17]. In Japan, illegal drug transactions conducted through popular flea market applications, social media, and auctions websites have been reported with most of the trades being OTC medications [18-20]. The pharmacological effects of OTC drug abuse or misuse have been characterized with, for example, dextromethorphan, codeine (opioid), diphenhydramine, and promethazine [21]. Specifically in Japan, OTC drug abuse is an emerging problem due to the availability of OTC drugs containing dihydrocodeine [21]. Harms associated with the abuse of such OTC drugs have been indicated to cause serious mental and physical effects, including symptoms of poisoning and dependence, hallucinations, euphoria, dissociative states, and even death [22-25]. Recognizing these risks, an effort has been reportedly made to detect individuals who potentially abuse OTC drugs from consumer-generated media in Japan, where users can generate content by posting on social media [26].

However, as a newly emerging trend, we previously discovered a unique and preliminary black market based on X (formerly Twitter) in Japan, where individuals engage in the trade of majorly prescription medications, instead of OTC medications, with a specific term, “Okusuri Mogu Mogu” [20]. “Okusuri” refers to medications, and “Mogu Mogu” is used as an onomatopoeia for eating or biting in Japanese. This specific term is local in Japan and is unique enough to be distinguished by other transaction keywords in that most of the traded medications are psychiatric prescription medications. Indeed, to assess the unique attributes of illicit trading using this specific term, the Japanese government has recently funded domestic investigative research [27].

Hence, monitoring of the dynamic methods of illicit trading should routinely be performed. This study extends our previous effort to elucidate illicit drug trading using the aforementioned specific term, especially to characterize what kind of medications are traded by whom and with what relative keywords, which were previously unknown. Herein, we report our latest findings for the “Okusuri Mogu Mogu” trend and an analysis of hashtags to investigate individual behaviors associated with X posts.

## Methods

### Study Sample

In this cross-sectional study, we systematically investigated posts published on X (formerly Twitter) using the search term “Okusuri Mogu Mogu” in accordance with a previously described approach [20]. We selected this term because several surveillance and research studies reported that it is used by individuals to trade medications. One surveillance activity in particular was led by grants from the Japanese regulatory authority, the Ministry of Health, Labour and Welfare (MHLW) [27]. Moreover, an organization was commissioned by the MHLW to monitor this term through their original X account to alert X users [28].

### Data Collection and Categorization

We investigated X posts by inputting the term “Okusuri Mogu Mogu” in Japanese in the X search field without adding a hashtag. Posts published between September 18 and October 1, 2022, at Japan Standard Time were collected in an Excel (Microsoft Corp) spreadsheet for data analysis. Extracted post data included the date of posting, whether a hashtag was used for “Okusuri Mogu Mogu,” whether the post had accompanying images, the categorization of the post (ie, buying, selling, self-administration, heads-up posts for illegal trading, and unknown or unclassified), and the name of pharmaceutical products mentioned in the posts or images. Independent counts of medication names and hashtags were processed, which yielded overlaps. All posts were extracted within 1 week of the date of posting as users can delete posts shortly after posting.

### Data Cleaning

Posts were distributed to 2 individual blinded researchers’ groups, and they categorized each extracted post and compared the categorization between groups. Posts that were detected for only 1 group were excluded from the analysis, as were posts that had inconsistent categorization between groups.

### Enumeration and Categorization of Posts Related to Buying or Selling of Drugs

Posts related to buying or selling of pharmaceutical products were further analyzed. We followed the categorization of criteria that have been previously reported [20]. The classification criteria are shown in Table S1 in Multimedia Appendix 1. We enumerated the drugs mentioned in these posts using a text-mining tool that was available on the web [29]. If the named drugs were not mentioned using their official generic name but instead referenced using an abbreviation or a brand name, each term was allocated to a specific generic name based on author consensus before enumeration. We also categorized the detected pharmaceutical drugs using the Anatomical Therapeutic Chemical (ATC) classification. ATC codes and therapeutic categories were investigated using the KEGG (Kyoto Encyclopedia of Genes and Genomes) BRITE database [30].

### Enumeration of Drugs in Each Category of Posts

Mentions of product names in posts that implied self-administration were also enumerated as described above. We then enumerated the drugs in each category of posts, including buying, selling, and self-administration, before sorting the counts from the most to the fewest number of mentions. Statistical analyses were carried out using Excel.

### Hashtag Classification

The number of hashtags in all the collected posts was determined except for the hashtag “Okusuri Mogu Mogu.” The extracted hashtags were further manually classified into 6 categories: medication name, mental disorder, self-harm, buying and selling, community formation, and other.

### Ethical Considerations

This cross-sectional observational study was performed only with publicly available data and did not involve any interventions or individual patient records. Thus, institutional review board approval and patients’ informed consent were not necessary.

## Results

### Extracting Posts for Analysis

**Figure 1:**
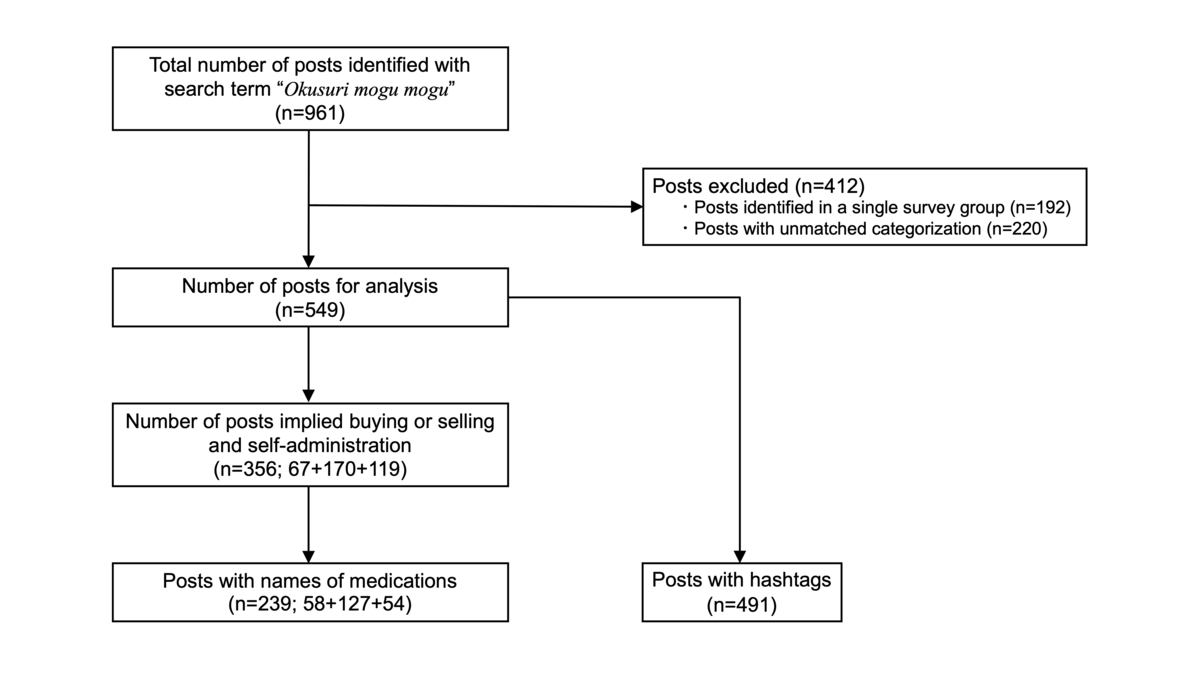
Flowchart for the inclusion of X (formerly Twitter) posts in the analysis. During the 2-week investigation period between September 18 and October 1, 2022, a total of 961 posts were identified. Posts identified only in a single survey group (n=192) and those that share unmatched categorizations between groups (n=220) were excluded from the analysis. Among the remaining 549 posts, 356 referenced buying (n=67) or selling (n=170) or self-administration (n=119) of medications. All the names of the mentioned medications were subjected to Anatomical Therapeutic Chemical–based categorization. Of the 356 posts that implied buying, selling, or self-administration of medication, 239 mentioned names of medications. Similarly, among the 549 posts in the analysis, 491 included at least 1 hashtag, and all hashtags were enumerated.

During the 2-week investigation period between September 18 and October 1, 2022, a total of 961 posts were identified using the search term “Okusuri Mogu Mogu.” These posts were grouped into 5 categories: buying, selling, self-administration, heads-up posts for illegal trading, and unknown or unclassified. To verify the categorization, researchers divided themselves into 2 independent groups and categorized the posts, followed by the category-matching of individual posts. Since posts of interest were not collected simultaneously, some of them (n=192) were identified only in a single group. All of these posts were excluded from the analysis. Posts that had unmatched categories between groups (n=220) were also excluded. The remaining 549 posts were included in the analysis (Figure 1).

### Categorization of Posts for Analysis

The characteristics and categorization of the 549 posts are shown in Table 1. Among these posts, 491 (89.4%) included the hashtag “Okusuri Mogu Mogu.” A total of 287 (52.3%) posts included the name of at least 1 medication, and 205 (37.3%) posts included visual images such as tablets, bottles, blister packs, and inventory lists of medications as part of sales promotions. Of note, over 40% of all posts were categorized as transactions of medications: 67 (12.2%) mentioned buying or an intent to purchase medications and 170 (31.0%) referenced selling, promoting, and attempts to give away medications.

Users also referenced “Okusuri Mogu Mogu” to manifest their digital presence. Instances of self-administration accounted for 21.7% (n=119) of posts, wherein users self-administered their medication before or after posting on X, or usage on a regular basis. Only 14 (2.6%) posts served as a heads-up regarding illegal transactions of medications to users. Around one-third of all posts (n=179, 32.6%) could not be classified or had no discernible meaning.

**Table 1:** Characteristics and categorization of posts for analysis (N=549)^a,b^.

Characteristics			Posts, n (%)
Posts with hashtag “Okusuri Mogu Mogu”			491 (89.4)
Posts that mentioned at least one medication name			287 (52.3)
Posts with images			205 (37.3)
**Categories**			
	Buying: posts related to hoping, trying and wanting to buy or get	67 (12.2)	
	Selling: posts related to selling, promotion of selling, and attempts to give away medications	170 (31.0)	
	Self-administration: posts indicating users’ drug administration, either before or after taking pills and regularly	119 (21.7)	
	Heads-up: posts from official or unofficial accounts that alert illegal medication trading	14 (2.6)	
	Others: unknown, unclassifiable, and other posts	179 (32.6)	

^a^Characteristics and categorization of 549 posts in X (formerly Twitter) for analysis, which were collected between September 18 and October 1, 2022.

^b^Detailed categorization criteria are described in Table S1 in Multimedia Appendix 1. We categorized posts in accordance with a previously published method [20].

### ATC Classification of Posts Indicating Medication Transactions

To understand what kinds of medications are prevalent on the SNS-based black market, we systematically counted and categorized the names of medications that were identified in 237 posts included in the buying (n=67) and selling (n=170) categories (Figure 1). The categorization was assigned in accordance with the ATC classification. Of these 237 posts, 185 (78.1%) contained at least 1 specific medication name, and 1041 distinct medication names were mentioned (Table S2 in Multimedia Appendix 2). The number of medications mentioned in the posts for each ATC category is shown in Table 2; herein, we used the same screening and categorization processes as those reported previously in a survey conducted in March 2021 so that we could compare changes in circumstances surrounding illicit trading with the keyword “Okusuri Mogu Mogu” [20]. Notably, when classified using the ATC classification, drugs that affect the nervous system dominated, constituting 82.1% of all mentioned medication names. This trend is consistent with that observed in a previous study [20]. However, relative to our earlier study, the average daily count of mentioned medication names was substantially higher, exhibiting a 5-fold increase overall and an 8-fold increase for medications that act on the nervous system. Along with this substantial upsurge in the mentions of medication names, the names of multiple medications that had not previously been mentioned were represented across diverse ATC categories in this study. These categories include drugs impacting the alimentary canal and metabolism, those impacting hematopoiesis and hematopoietic organs, dermatological drugs, musculoskeletal system drugs, and Kampo medications. In addition, a drug that has not been approved by the Japanese government was mentioned 7 times.

**Table 2:** Medications mentioned in posts that implied buying or selling, categorized on the basis of the Anatomical Therapeutic Chemical (ATC) classification.

Drug categories		September 18 to October 1, 2022 (N=1041; an average of 74.4 posts daily mentioning medications)^a^	March 1-8, 2021 (N=118; an average of 14.8 posts daily mentioning medications)^b^
[A] Alimentary tract and metabolism		36 (3.5)	1 (0.8)
[B] Blood and blood forming organs		11 (1.1)	0 (0)
[C] Cardiovascular system		1 (0.1)	0 (0)
[D] Dermatological		19 (1.8)	1 (0.8)
[G] Genitourinary system and sex hormones		8 (0.8)	3 (2.5)
[H] Systemic hormonal preparations, excluding sex hormones and insulins		0 (0)	0 (0)
[J] Anti-infectives for systemic use		11 (1.1)	0 (0)
[L] Antineoplastic and immunomodulating agents		0 (0)	0 (0)
[M] Musculoskeletal system		12 (1.2)	0 (0)
**[N] Nervous system**		855 (82.1)	107 (90.7)
	N02: analgesics	13 (1.3)	0 (0)
	N03: antiepileptics	119 (11.4)	4 (3.4)
	N05: psycholeptics	625 (60.0)	99 (83.9)
	N05A: antipsychotics	124 (11.9)	1 (0.8)
	N05B: anxiolytics	219 (21.0)	15 (12.7)
	N05C: hypnotics and sedatives	282 (27.1)	83 (70.3)
	N06: psychoanaleptics	87 (8.4)	4 (3.4)
	N07: other nervous system drugs	11 (1.1)	0 (0)
[P] Antiparasitic products, insecticides, and repellents		0 (0)	0 (0)
[R] Respiratory system		0 (0)	0 (0)
[S] Sensory organs		0 (0)	0 (0)
Kampo medicines		35 (3.4)	0 (0)
More than 2 classifications		22 (2.1)	2 (1.7)
No ATC classifications		14 (1.3)	3 (2.5)
Over-the-counter medications		10 (1.0)	1 (0.8)
Unapproved drugs in Japan		7 (0.7)	0 (0)

^a^Among 237 posts that implied transactions (67 for buying and 170 for selling), 185 (78.1%) contained at least 1 specific medication name, totaling up to 1041 distinct medication names mentioned in these posts. These medications were categorized using the ATC classification.

^b^For comparison, medication names collected from March 1-8, 2021, using the same screening and categorization processes, are been listed [20].

### Trends for Mentioned Medication Names According to Categories of Posts

We next investigated whether there are discrepancies in a series of medications mentioned across different categories of posts. As expected, drugs that affect the nervous system, such as flunitrazepam, zolpidem, and etizolam, were commonly observed across categories on comparing the top 10 lists of medication names present in each post category (Table 3). We also enumerated the mentions of medication names in the self-administration category (Table 3 and Table S3 in Multimedia Appendix 3). Interestingly, OTC drugs such as SS Bron and Medicon appeared more frequently in the self-administration category (odds ratio 23.6, 95% CI 6.93-80.15) than in the other transactional posts. This finding suggests that discrepancies may exist between transactional posts and self-administration posts in terms of mentions of medication names.

**Table 3:** Top 10 medication names for each post category^a^.

Rank	Categories					
	Selling		Buying		Self-administration	
	Generic name	Mentions, n	Generic name	Mentions, n	Generic name	Mentions, n
1	Flunitrazepam	72	Flunitrazepam	26	Flunitrazepam	13
2	Lorazepam	61	Zolpidem	21	SS Bron^b^	9
3	Risperidone	56	Etizolam	19	Zolpidem	7
4	Zolpidem	53	Methylphenidate	7	Etizolam	6
5	Etizolam	44	Eszopiclone	5	Medicon^b^	6
6	Clonazepam	44	Triazolam	4	Pentobarbital	5
7	Sodium valproate	38	Pentobarbital	3	Lemborexant	5
8	Bromazepam	33	Lorazepam	3	Olanzapine	4
9	Clotiazepam	29	Zopiclone	2	Clonazepam	4
10	Brotizolam	28	Brotizolam	2	Pregabalin	4
Total	N/A^c^	935	N/A	106	N/A	129

^a^Among 549 posts for analysis, 356 indicated either buying (n=67), selling (n=170), or self-administration (n=119). Furthermore, 86.6% (58/67) of posts were categorized as buying, 74.7% (127/170) of them were categorized as selling, and 45.4% (54/119) of them were categorized as self-administration, including at least 1 medication name. These medication names were enumerated and the top 10 in these lists are shown.

^b^Over-the-counter drugs.

^c^N/A: not applicable.

### Hashtag Analysis

Finally, we analyzed all the hashtags present in the 549 posts that were considered. A total of 491 posts in the sample included hashtags. In addition to the hashtag “Okusuri Mogu Mogu,” 866 hashtags were detected. We classified these hashtags into 6 categories: medication name, mental disorder, self-harm, buying and selling, community formation, and other (Table 4). As anticipated, numerous psychiatric drugs were explicitly mentioned, and correspondingly, a substantial number of the identified hashtags were linked to symptoms related to mental disorders and self-inflicted harm. Within the transaction-related categories, the abbreviation “Letapa,” which refers to “Letter Pack,” a postal service envelope manufactured by Japan Post Co, Ltd, ranked prominently, implying its use as a transaction method for the referenced medications. In addition to psychiatric keywords, hashtags indicative of “community formation” that encompassed endeavors to establish connections on the SNS were prevalent.

**Table 4:** Top 5 hashtags appearing in the analyzed posts^a^ for each hashtag category.

Hashtag		Hashtags, n
**Medication name**		
	Depas (generic name: etizolam)	30
	Silece (generic name: flunitrazepam)	24
	Myslee (generic name: zolpidem)	16
	Halcion (generic name: triazolam)	12
	Medicon (generic name: dextromethorphan)	10
**Mental disorder**		
	Insomnia	15
	Sleep disorders	8
	Attachment disorder	5
	Eating disorder	4
	Bulimia nervosa	4
	Mental disorders	4
**Self-harm**		
	Overdose	17
	Wrist cut	8
	*Amuka*	7
	Self-harm	5
	Self-harming account	2
**Buying and selling**		
	*Letapa*	13
	*Okusuri Yuzurimasu*	10
	Eradicate fraudsters	7
	Fraud	3
	Private import	3
**Community formation**		
	*Yami-aka-san to Tsunagaritai*	24
	*Yami-aka*	22
	*Menhera*	14
	*Menhera-joshi*	10
	*Arasa-joshi*	9
	*Ura-aka-joshi*	9
	*Okusuri charm*	9

^a^Original posts were in Japanese and were translated to English. Total number of hashtags was 866 among 491 analyzed posts. “Okusuri Mogu Mogu” was excluded because all analyzed posts included this term. Hashtags in *italics* are the original Japanese hashtags and had the following definitions: *Amuka*: arm + cut (indicates self-harm by cutting the arm); *Letapa*: abbreviated form of “Letter Pack” (mail service of Japan Post Co, Ltd); *Okusuri Yuzurimasu*: offering medication; *Yami-aka-san to Tsunagaritai*: a desire to connect with others who share similar struggles with “*Yami*,” indicating something on the dark side; *Yami-aka*: sharing of personal struggles related to mental health through social networking service accounts; *Menhera*: based on a term for mental health, referencing individuals who openly discuss their mental health struggles on the internet or people who are perceived as being clingy; *Menhera-joshi*: women who have “*menhera*” and “*joshi*” is a term for females, including both women and girls; * Arasa-joshi*: women in their 30s; Arasa: individuals who are around 30 years old; *Ura-aka-Joshi*: women who have secret or private accounts; *Okusuri charm*: accessories traded on social networking services that are crafted from blister packs used for prescription medications.

## Discussion

### Principal Findings

#### Background

In this cross-sectional study, we used the specific search term “Okusuri Mogu Mogu” to identify posts published by individual users of X (formerly Twitter) in Japan. These posts were published during a 2-week study period in late September 2022, and concerned different types of transactions, majorly involving prescription medications (95.6%-99.0%). We found an increasing diversity in the mentioned medications, encompassing Kampo medications, OTC drugs, and medications that have not received approval for use in Japan. Further systematic analysis of the mentioned medication names in each post category revealed a dominance of nervous system drugs among both trading users and individuals who expressed self-dosage of medication, and highlighted the extensive self-administration of OTC drugs by individuals. Our hashtag analysis, which sought to understand user behaviors, supported the possibility that these individuals could have relevant mental disorders or mental concerns and indicated attempts to foster distinctive communities among these users.

#### Transactions

The high frequency of medication names we observed in posts related to selling could be related to reposts and inventory lists posted by sellers (Table 3). Meanwhile, the lower frequency of posts related to buying might be due to the exchange of communication interfaces used to acquire drugs. Some posts indicated that discussions concerning details about trading may have switched from public posts to alternative apps or direct messaging.

The number of posts related to medication transactions increased drastically over a span of 18 months since our prior study [20]. Concurrently, the number of mentions increased for medications that were not referenced in the previous study. One reason for this increase might be due to the growing complexity of selling strategies. In fact, posts featuring lists of medications and images of inventory lists were identified in this survey, and such lists may enable traders to deal in medications systematically. Such image-based inventory lists can be a new strategy to protect traders from account suspension. Other traits we noted were prompt responses of sellers to market changes. For instance, we newly detected Lemborexant, a hypnotic that received approval in 2020 for which there has been increased marketing activity, and Medicon, an OTC cough suppressant that contains dextromethorphan and has been marketed since 2021. Neither Lemborexant nor Medicon were identified in our previous study in March 2021. Taken together, these results support the increased complexity and strategic sales techniques used by dealers.

#### Concerns About OD of OTC Medications

We found that OTC medications were mentioned with higher frequency in posts in the self-administration category relative to those in the trading categories, suggesting that such OTC medications are not traded on the “Okusuri Mogu Mogu”–based black market, but are instead obtained by individuals themselves. This finding suggests that individuals who consume OTC medications are clearly distinct from those who trade in prescription medications, even though both types of users included the term “Okusuri Mogu Mogu.” We note 2 important implications regarding self-administration of OTC medications.

First, the risk of OD from OTC medications is apparent, especially OD due to the use of antitussive OTC medications that include codeine, dihydrocodeine, and methylephedrine [6,8,31]. Indeed, our results show that SS Bron, which contains dihydrocodeine, is the most frequently administered OTC medication among individuals (Table 3). To reduce the risk of OD caused by OTC medications, since April 1, 2023, the MHLW expanded their specified range of “pharmaceuticals that are prone to abuse or misuse” to include 6 specific compounds present in OTC medications: ephedrine, codeine, dihydrocodeine, bromovalerylurea, pseudoephedrine, and methylephedrine [32]. This policy modification expanded the scope of designated products and emphasizes the efforts of the MHLW to decrease the rate of OD caused by OTC medications. Additional studies are needed to evaluate whether such pharmacovigilance policy changes do, in fact, affect OD rates.

Second, regulatory endeavors by agencies may be insufficient to change OD incidence. Such regulatory stances can potentially result in a protracted, contrived pursuit of illicit drug traders. In this study, the second-most prevalent OTC medication mentioned by users self-administering medication was Medicon (Table 3), which has been marketed since 2021. Despite the risk of OD caused by its active component dextromethorphan, Medicon was not included in the MHLW list of “pharmaceuticals that are prone to abuse or misuse” [32], perhaps because of a lack of information due to its relatively recent appearance in the market. Our findings provide 2 perspectives. First, the digital age of SNSs can easily allow individuals to evade regulations; and second, OD caused by OTC drugs differs from dependency on narcotics and stimulants. Purchasing OTC drugs is legal; hence, complete prohibition through regulatory measures would be difficult. For individuals for whom the risk of OD could increase through the use of SNSs, contact with self-help groups and simple acknowledgment that such users exist in the actual world, rather than a regulatory crackdown, could be an effective strategy to reduce the risk of OD.

#### Community-Forming Behaviors

Our study sample indicates that users are beginning to interact with each other and form distinct communities, which is consistent with previous research [33]. The most dominant hashtag in the community formation category was “*Yami-aka-san to Tsunagaritai*,” which reflects a direct intention to interact among users: “*Yami-aka-san*” is associated with the sharing of personal struggles related to mental health, and “*Tsunagaritai*” literally expresses the desire of an individual to connect with someone. Such communication-intended posts were not obvious in our previous investigation.

Additionally, user interactions went beyond hashtags. An intriguing fashionable accessory, termed “Okusuri Charm,” is crafted from blister packs used to package prescription drugs, and recently emerged in posts including relevant hashtags [34]. Interactions through such accessories is now a movement, indicating another endeavor to form a community.

Such community formation within SNSs may represent traits of users, who have fragile connections with the real world and are potentially isolated, particularly since more than a few hashtags related to mental disorders or mental concerns accompanied the use of “Okusuri Mogu Mogu.” Unfortunately, in a highly anonymous community, sellers of medications can take advantage of the potential weakness of these users to commit fraud, which was identified in our hashtag analysis.

### Limitations

Our study has several limitations. First, posts collected in this study were published within a 2-week period, and the number of posts analyzed was relatively small. Moreover, we considered only a single search term “Okusuri Mogu Mogu.” Some posts could not be included since users can delete their posts as well as their account, and posts from private accounts could not be collected. Thus, our results may underestimate the actual situation associated with illegal trade of medications. Second, our results should carefully be interpreted since they were based on manual investigation of posts. For instance, many posts were excluded because they could not be categorized, and the categorization itself was based on researchers’ individual inspections. Indeed, 22.9% (220/961) of posts were excluded from the analysis due to classification discrepancies between raters, indicating that posts with ambiguous classification might still remain (Figure 1). Further studies with regular web scraping or machine learning–based categorization are needed to further elucidate individual behaviors related to medication trading [35].

### Conclusions

In this study, we elucidated the current situation surrounding illegal prescription drug transactions facilitated by SNSs, by scrutinizing X (formerly Twitter) posts that contained the term “Okusuri Mogu Mogu.” Our cross-sectional study highlights the escalating quantity and diverse array of the mentioned names of medications and the increasing complexity of strategies to allow transactions to occur. We also unveiled distinctive characteristics among users around such black markets for illegal medication trading. A hashtag analysis indicated that users may use SNSs to foster communication and form a sense of communal affiliation among users. From a pharmacovigilance perspective, it is judicious for the authorities to enhance public awareness by instituting stringent measures to prevent illegal transactions of prescription medications. However, a regulatory stance alone may inadvertently reinforce cohesiveness within the confines of a closed community. Social approaches, such as self-help groups, which could direct individuals to appropriate medical or psychiatric resources would represent viable pathways to support socially vulnerable SNS users.

## Appendix

Multimedia Appendix 1 Classification criteria of posts and their categorization (n=549).

Multimedia Appendix 2 A whole list of the number of medication names among tweets implied buying or selling. Kampo medication is represented in light blue and OTC medication in green. OTC: over the counter.

Multimedia Appendix 3 A whole list of medication names and no. of cases mentioned in the posts that implied trading. Kampo medication is represented in light blue, OTC medication in green, nationally unapproved drugs in purple, and a discontinued drug in red, respectively. OTC: over the counter.

## Abbreviations

ATC: Anatomical Therapeutic Chemical
KEGG: Kyoto Encyclopedia of Genes and Genomes
MHLW: Ministry of Health, Labour and Welfare
OD: overdose
OTC: over-the-counter
SNS: social networking service

## Data Availability

Data generated or analyzed during this study are included in this article and Multimedia Appendices 1-3. More detailed data are available from the corresponding author on reasonable request.
