# Supplementary material for: Illicit Trade of Prescription Medications Through X (Formerly Twitter) in Japan: Cross-Sectional Study
Source: JMIR Form Res. 2024 May 28;8:e54023. doi: 10.2196/54023 (PMC11167319; doi:10.2196/54023)
Supplement: Multimedia Appendix 1 [file formative_v8i1e54023_app1.pdf]

Table S1. Classification criteria of posts and their categorization (n=549).

Categories	Criteria	Proportion
Buying	Posts that mentioned buying, getting and attempt to buying or getting medications were included.	67 (12.2%)
Selling	Posts that mentioned selling, advertiseing, and giving medications to other users were included. Posts that mentioned or implied prices, transportation methods, discounting, and promoting their inventory user's of medications were also included.	170 (31.0%)
Self-administration	Posts that mentioned user's drug administration, either before or after taking pills were included. Posts that implied their daily or regular self-administration was also included.	119 (21.7%)
Heads up for illegal trading	Posts that head up to users as illegal transactions of medications. Posts from an official account commissioned by the Japan's Ministry of Health, Labour and Welfare were also included.	14 (2.6%)
Others	Posts that could not be classified, such as the one with no meanings or had no discernible meanings.	179 (32.6%)
