# Supplementary material for: Illicit Trade of Prescription Medications Through X (Formerly Twitter) in Japan: Cross-Sectional Study
Source: JMIR Form Res. 2024 May 28;8:e54023. doi: 10.2196/54023 (PMC11167319; doi:10.2196/54023)
Supplement: Multimedia Appendix 2 [file formative_v8i1e54023_app2.pdf]

Table S2. A whole list of the number of medication names among tweets implied buying or selling.

Name of medications	No. of cases	ATC code
Flunitrazepam	98	N05CD03
Zolpidem	74	N05CF02
Lorazepam	64	N05BA06
Etizolam	63	N05BA19
Risperidone	56	N05AX08
Clonazepam	44	N03AE01
Sodium valproate	38	N03AG01
Bromazepam	34	N05BA08
Brotizolam	30	N05CD09
Clotiazepam	29	N05BA21
Pregabalin	26	N03AX16
Levomepromazine	25	N05AA02
Triazolam	24	N05CD05
Quetiapine	23	N05AH04
Rebamipide	21	A02BX14
Methylphenidate	21	N06BA04
Tofisopam	19	D01254
Trazodone	19	N06AX05
Alprazolam	19	N05BA12
Magnesium oxide	19	A02AA02 A06AD02 A12CC10
Suvorexant	18	N05CM19
Lemborexant	16	N05CM21
Venlafaxine	12	N06AX16
Acotiamide	12	No ATC code
Linaclotide	11	A06AX04
Mecobalamin	11	B03BA05
Clarithromycin	11	J01FA09
Loxoprofen	11	M02AA31
Mirtazapine	11	N06AX11
Betahistine	11	N07CA01
Ethyl Loflazepate	10	N05BA18
Eszopiclone	10	N05CF04
Tramadol	9	N02AX02
Phenobarbital	9	N03AA02
**Keishibukuryogan**	**9**	**Kampo**
**Goreisan**	**9**	**Kampo**
**Shakuyakukanzoto**	**9**	**Kampo**
Sildenafil	8	G04BE03
Pemoline	8	N06BA05
Asenapine	7	N05AH05
Duloxetine	7	N06AX21
Zaleplon (unapproved)	7	unapproved drug
**Bofutsushosan**	**6**	**Kampo**
Olanzapine	5	N05AH03
Pentobarbital	4	N05CA01
Dextromethorphan	4	R05DA09
Acetaminophen	3	N02BE01
Chlorpromazine	3	N05AA01
Nitrazepam	3	N05CD02
Escitalopram	3	N06AB10
Famotidine	2	A02BA03
Carbamazepine	2	N03AF01
Lormetazepam	2	N05CD06
Zopiclone	2	N05CF01
Imipramine	2	N06AA02
Promethazine	2	D04AA10 R06AD02
Caffeine and sodium benzoate	2	No ATC code
**Restamin® (OTC)**	**2**	**OTC**
**Gaster® (OTC)**	**2**	**OTC**
**SS Bron® (OTC)**	**2**	**OTC**
Metoclopramide	1	A03FA01
Domperidone	1	A03FA03
Guanfacine	1	C02AC02
Tizanidine	1	M03BX02
Morphine	1	N02AA01
Sulpiride	1	N05AL01
Lithium carbonate	1	N05AN01
Zotepine	1	N05AX11
Aripiprazole	1	N05AX12
Brexpiprazole	1	N05AX16
Quazepam	1	N05CD10
Clomipramine	1	N06AA04
Paroxetine	1	N06AB05
Fluvoxamine	1	N06AB08
Modafinil	1	N06BA07
Diclofenac Sodium	1	M01AB05 M02AA15
**Kakkonto**	**1**	**Kampo medication**
**Tokishakuyakusan**	**1**	**Kampo medication**

OTC = Over the counter.
