# Supplementary material for: Illicit Trade of Prescription Medications Through X (Formerly Twitter) in Japan: Cross-Sectional Study
Source: JMIR Form Res. 2024 May 28;8:e54023. doi: 10.2196/54023 (PMC11167319; doi:10.2196/54023)
Supplement: Multimedia Appendix 3 [file formative_v8i1e54023_app3.pdf]

Table S3. A whole list of the number of medication names among tweets implied buying or selling.

Name of medications	No. of cases	ATC code	Name of medications	No. of cases	ATC code
Flunitrazepam	98	N05CD03	Asenapine	7	N05AH05
Zolpidem	74	N05CF02	Duloxetine	7	N06AX21
Lorazepam	64	N05BA06	Zaleplon (unapproved)	7	unapproved drug
Etizolam	63	N05BA19	**Bofutsushosan**	**6**	**Kampo**
Risperidone	56	N05AX08	Olanzapine	5	N05AH03
Clonazepam	44	N03AE01	Pentobarbital	4	N05CA01
Sodium valproate	38	N03AG01	**Medicon ® (OTC)**	**4**	**OTC**
Bromazepam	34	N05BA08	Acetaminophen	3	N02BE01
Brotizolam	30	N05CD09	Chlorpromazine	3	N05AA01
Clotiazepam	29	N05BA21	Nitrazepam	3	N05CD02
Pregabalin	26	N03AX16	Escitalopram	3	N06AB10
Levomepromazine	25	N05AA02	Famotidine	2	A02BA03
Triazolam	24	N05CD05	Carbamazepine	2	N03AF01
Quetiapine	23	N05AH04	Lormetazepam	2	N05CD06
Rebamipide	21	A02BX14	Zopiclone	2	N05CF01
Methylphenidate	21	N06BA04	Imipramine	2	N06AA02
Tofisopam	19	D01254	Promethazine	2	D04AA10 R06AD02
Trazodone	19	N06AX05	Caffeine and sodium benzoate	2	No ATC code
Alprazolam	19	N05BA12	**Restamin® (OTC)**	**2**	**OTC**
Magnesium oxide	19	A02AA02 A06AD02 A12CC10	**Gaster® (OTC)**	**2**	**OTC**
Suvorexant	18	N05CM19	**SS Bron® (OTC)**	**2**	**OTC**
Lemborexant	16	N05CM21	Metoclopramide	1	A03FA01
Venlafaxine	12	N06AX16	Domperidone	1	A03FA03
Acotiamide	12	No ATC code	Guanfacine	1	C02AC02
Linaclotide	11	A06AX04	Tizanidine	1	M03BX02
Mecobalamin	11	B03BA05	Morphine	1	N02AA01
Clarithromycin	11	J01FA09	Sulpiride	1	N05AL01
Loxoprofen	11	M02AA31	Lithium carbonate	1	N05AN01
Mirtazapine	11	N06AX11	Zotepine	1	N05AX11
Betahistine	11	N07CA01	Aripiprazole	1	N05AX12
Ethyl Loflazepate	10	N05BA18	Brexpiprazole	1	N05AX16
Eszopiclone	10	N05CF04	Quazepam	1	N05CD10
Tramadol	9	N02AX02	Clomipramine	1	N06AA04
Phenobarbital	9	N03AA02	Paroxetine	1	N06AB05
**Keishibukuryogan**	**9**	**Kampo**	Fluvoxamine	1	N06AB08
**Goreisan**	**9**	**Kampo**	Modafinil	1	N06BA07
**Shakuyakukanzoto**	**9**	**Kampo**	Diclofenac Sodium	1	M01AB05 M02AA15
Sildenafil	8	G04BE03	**Kakkonto**	**1**	**Kampo medication**
Pemoline	8	N06BA05	**Tokishakuyakusan**	**1**	**Kampo medication**

OTC = Over the counter.
